# Automated Monitoring of Suicidal Adolescents’ Digital Media Use: Qualitative Study Exploring Acceptability Within Clinical Care

**DOI:** 10.2196/26031

**Published:** 2021-09-15

**Authors:** Candice Biernesser, Jamie Zelazny, David Brent, Todd Bear, Christina Mair, Jeanette Trauth

**Affiliations:** 1 Department of Psychiatry University of Pittsburgh Pittsburgh, PA United States; 2 School of Nursing University of Pittsburgh Pittsburgh, PA United States; 3 Department of Behavioral and Community Health Sciences University of Pittsburgh Pittsburgh, PA United States

**Keywords:** adolescents, parents, digital media, social media, technology, monitoring, suicide prevention, implementation in clinical care, natural language processing, qualitative

## Abstract

**Background:**

Monitoring linguistic cues from adolescents’ digital media use (DMU; ie, digital content transmitted on the web, such as through text messages or social media) that could denote suicidal risk offers a unique opportunity to protect adolescents vulnerable to suicide, the second leading cause of death among youth. Adolescents communicate through digital media in high volumes and frequently express emotionality. In fact, web-based disclosures of suicidality are more common than in-person disclosures. The use of automated methods of digital media monitoring triggered by a natural language processing algorithm offers the potential to detect suicidal risk from subtle linguistic units (eg, negatively valanced words, phrases, or emoticons known to be associated with suicidality) present within adolescents’ digital media content and to use this information to respond to alerts of suicidal risk. Critical to the implementation of such an approach is the consideration of its acceptability in the clinical care of adolescents at high risk of suicide.

**Objective:**

Through data collection among recently suicidal adolescents, parents, and clinicians, this study examines the current context of digital media monitoring for suicidal adolescents seeking clinical care to inform the need for automated monitoring and the factors that influence the acceptance of automated monitoring of suicidal adolescents’ DMU within clinical care.

**Methods:**

A total of 15 recently suicidal adolescents (aged 13-17 years), 12 parents, and 10 clinicians participated in focus groups, qualitative interviews, and a group discussion, respectively. Data were recorded, transcribed, and analyzed using thematic analysis.

**Results:**

Participants described important challenges to the current strategies for monitoring the DMU of suicidal youth. They felt that automated monitoring would have advantages over current monitoring approaches, namely, by protecting web-based environments and aiding adolescent disclosure and support seeking about web-based suicidal risk communication, which may otherwise go unnoticed. However, they identified barriers that could impede implementation within clinical care, namely, adolescents’ and parents’ concerns about unintended consequences of automated monitoring, that is, the potential for loss of privacy or false alerts, and clinicians’ concerns about liability to respond to alerts of suicidal risk. On the basis of the needs and preferences of adolescents, parents, and clinicians, a model for automated digital media monitoring is presented that aims to optimize acceptability within clinical care for suicidal youth.

**Conclusions:**

Automated digital media monitoring offers a promising means to augment detection and response to suicidal risk within the clinical care of suicidal youth when strategies that address the preferences of adolescents, parents, and clinicians are in place.

## Introduction

### Background

There is growing public health interest in strategies for monitoring the digital media use (DMU) of youth at risk for suicide, the second leading cause of death among adolescents [1]. DMU, which we define as digitized content transmitted on the web, for example, through social media or text messages, can offer adolescents a number of important benefits, such as social support and connectedness [2,3]. However, youth at risk for suicide may be especially vulnerable to negative digital media experiences that can contribute to changes in mood and mental state [4,5]. Compared with youth in the general population, adolescents facing mental health challenges are more likely to be exposed to explicit or triggering content such as images or descriptions of self-harm behavior through their DMU [3], report negative experiences such as cyberbullying [6], and engage in problematic internet use (ie, difficulty in controlling use that has negative consequences in daily life) [7,8]. Although there is potential for greater vulnerability to negative digital media experiences, there are currently no evidence-based approaches for monitoring the DMU of youth at risk for suicide.

Monitoring linguistic cues on DMU that could denote suicidal risk could prove especially advantageous in clinical care of suicidal adolescents. Suicidal disclosures are frequent within web-based spaces, perhaps even more so than through in-person communication [9]. Typical assessment practices within clinical settings rely heavily on the subjective reporting of suicidal thoughts and behaviors [10]. Patient self-report, although useful, is limited in predicting suicidal risk [10]. Patients vary in their ability and willingness to provide information about suicidal intent based on a variety of factors, including lack of insight, a wish to avoid more restrictive treatment, or a desire to thwart detection to carry out suicidal plans [10]. The use of algorithms to guide clinicians’ conceptualization of risk is recommended to advance the current methods of suicide risk assessment [11]. Given the high rate of adolescent DMU [12], an algorithm that identifies language indicative of suicidal risk from adolescents’ DMU could be a beneficial supplement to the available assessment methods.

Advances in computer science and language analytic methods, such as natural language processing (NLP), allow for large amounts of textual data to be collected and analyzed [13], such as the high volume of web-based content produced by adolescents. Using a variety of analytic methods, NLP can evaluate the frequency and structure of linguistic units, features of interpersonal awareness, and emotional and psychological states [13]. NLP has shown the capacity to detect subtle indicators of suicidal risk within digital media content over time [14,15], highlighting its potential to enable an automated approach for monitoring suicidal risk.

### Objectives

A critical issue for the implementation of an automated digital media monitoring approach to detect suicidal risk is its acceptability within clinical care of youth identified to be at high risk for suicidal behavior, who have unique and acute needs for monitoring. Through data collection among youth who have experienced recent suicidal thoughts or behaviors, parents, and clinicians, this study examined (1) the current context of digital media monitoring for recently suicidal adolescents and (2) the factors that influence the acceptance of automated monitoring of suicidal adolescents’ DMU within clinical care.

## Methods

### Sample

A purposive sample [16] of adolescents aged 13-18 years in treatment for recent suicidal thoughts or behaviors, parents of adolescent patients, and mental health clinicians from an intensive outpatient program (IOP) at an academic medical center in Pennsylvania were invited to participate in this study. Of 254 youth within the patient population, 202 (79.5%) reported their biological sex as female and 52 (20.5%) as male. Of 244 patients who offered information on their race and ethnicity, 199 (81.6%) reported being White, 24 (9.8%) Black, 8 (3.3%) Asian, 8 (3.3%) Multi-racial, and 5 (2%) Hispanic. Of 217 patients who reported their sexual identity, 95 (43.8%) identified as 100% heterosexual and 122 (56.2%) as bisexual, mostly gay, 100% gay, or they did not know. Of 210 patients who reported their gender identity, 26 (12.4%) identified as transgender, genderqueer, nonbinary, or other gender identity.

### Data Collection

The first and second authors were introduced to adolescents and parents by clinicians to discuss the study. Adolescents and parents were not recruited as dyads, although 9 parents also had children who participated in the study. Interested adolescents participated in 3 focus groups (n=15). Focus groups ranged in size from 3 to 6 members, based on the patients enrolled within the IOP at the time of enrollment. Interested parents participated in interviews (n=12). Research visits were conducted separately for youth and parent participants. Parents provided informed consent, and the adolescents provided assent. Clinicians (n=10) were recruited via an email invitation and consented to participate in a group discussion, a form of qualitative data collection that derives shared meanings from people who have common experiences [17]. This study was approved by the University of Pittsburgh Institutional Review Board.

Data collection for patients and parents occurred between January and July 2018, and subsequent data collection for clinicians was conducted in January 2020. Appointments were conducted in person at research staff offices for adolescents and clinicians and either in person or via phone for parents based on their availability. All participants received an incentive of US $25. Data collection focused on experiences with digital media monitoring and perspectives on an automated approach to monitoring. To aid the recollection of mediation strategies deployed, parents completed a brief questionnaire via Qualtrics (version XM of Qualtrics) of selected items from the survey on Teens, Parents, and Digital Monitoring by the Pew Research Center [18]; subsequently, they were asked to expand upon the strategies they reported during interviews.

Guides were developed to facilitate semistructured discussion. The conversations remained open to topics most salient to the participants, which facilitated the spontaneous generation of themes. This approach increases the validity of experiential data collection [19]. The first and second authors conducted focus groups and the group discussion, and the first author conducted parent interviews. The inclusion of participants continued until saturation was reached [20].

### Data Analysis

Focus groups, interviews, and the group discussion were audiotaped, transcribed, and coded using NVivo, version 12 (QSR International) [21]. Data were analyzed using a thematic analysis approach designed by Braun and Clark [22], a recommended approach for applied health research [23]. Data were reviewed independently by the first and second authors using a codebook, which was created by the first author based on the interview script and revised to include emergent codes. Additions of new codes, changes in code definitions, and coding discrepancies were reviewed by consensus among the research team. Responses to the parent questionnaire were used to conceptualize the interview themes.

## Results

### Context for Digital Media Monitoring

#### Adolescent Focus Groups

Focus groups were conducted with 15 youth aged 13-17 years (mean 15.1, SD 1.6), 7 of whom were female, 5 of whom were male, and 2 of whom reported other gender identities. During the focus groups, adolescents acknowledged that DMU had positive influences but also mentioned that DMU negatively impacted their mental health and contributed to suicidal thoughts. However, most adolescents had mixed feelings toward the monitoring of their DMU, that is, they believed there should be a balance between their need for protection and for free expression and privacy. Some adolescents expressed moral opposition to monitoring, noting that it was an infringement of their personal freedoms. Likening it to having personal phone conversations endlessly recorded, they felt that monitoring was an invasion of privacy. Although others did not take a moral stance to monitoring, they reported valuing the ability to autonomously identify with a group of like-minded others on digital media. Some described having “Finstas” or secondary Instagram accounts. Although they often presented an idealized version of themselves on their primary accounts (eg, depicting a happy or successful persona), they used these secondary accounts to authentically express themselves to a limited number of trusted friends. They felt that monitoring these private conversations would diminish their ability to be themselves and limit their opportunities for peer support. All youth, even those who expressed a high degree of hesitance toward monitoring, agreed that monitoring of DMU is necessary when safety is a concern, for example, when self-harm is disclosed. They agreed that monitoring is especially important for younger children, who are more easily influenced and have a greater need for protection from negative experiences with DMU than adolescents.

Most adolescents said their parents did not monitor their DMU, and those who were monitored were strongly dissatisfied with their parents’ chosen monitoring methods. Adolescents consistently reported that they had never or infrequently discussed their DMU with their therapist, although they felt doing so would be helpful. Some youth were concerned about burdening their therapist with issues that felt inconsequential, such as getting enough *likes* or followers. They described apprehension in initiating these conversations and wanted prompting from therapists:

I think if it bothered me a lot, I wouldn’t be able to tell him...at all. Like I would have to be asked a question pertaining to it. I probably wouldn’t talk about it out of the blue.

#### Parent Interviews

Interviews were conducted with 12 parents, three-quarters of whom were mothers (N=9) and one-quarter were fathers (N=3). The parents’ mean age was 49.3 years (SD 4.2). During interviews, parents universally reported engaging in monitoring to protect their children. Parents reported feeling a strong obligation to monitor because they felt that negative digital media experiences (eg, cybervictimization) could trigger their children to have depressed or suicidal thoughts. Parents used a variety of strategies to mediate their children’s DMU (Table 1). Parents desired help in mediating their at-risk adolescents’ DMU, which they consistently reported took an emotional toll on them. They found it challenging to weigh the perceived benefit of mediation against the consequences of parent-child conflict and reducing prosocial digital interactions, which contributed to a sense of powerlessness:

I feel like, oh my gosh, it’s going to be my fault if she gets suicidal...So, I hate her phone, and I’m really frustrated at the lack of being able to truly control what she does.

**Table 1:** Parents’ strategies for mediating adolescents’ digital media use (N=12).

Mediation strategies and strategies parents used		Parents, n (%)	Successes	Challenges	Quote from a parent
**Co-use: parents engaging in DMU^a^ with their child**			Offered unique insights into adolescents’ emotional state and successful when other family members (eg, adult siblings) were engaged	Limited by adolescents’ use of multiple accounts that are not known to the parent	“It started out with- we said we had to have access to- we had to be not blocked. We had to be friends with her, so that we could see when she did post things. Then we noticed that she was having more than one account and we were friends with her on one account, but we were not friends with her on another account even though it existed. And, the school district actually called us and said that there were some things on there that were disturbing.”
	Friends with child on Facebook	4 (33)			
	Friends with child on Twitter	1 (8)			
	Friends with child on other social media platforms	5 (42)			
**Restriction: restriction of an adolescents’ DMU using social rules or technical means**			Removing phones from their child’s possession before bed was viewed as helpful to adolescents’ sleep, and blocking or filtering was considered useful for reducing content for mature audiences	Resulted in parent-child conflict, contributed to use behind parents’ back, restricted access to supportive friends, limited digital literacy impacted parents’ ability to use technical restrictions, and youth circumvented parental controls	“Like, I put parental controls on her phone, and she knows how to break into them and change them, and I feel very powerless a lot that all the monitoring that I know how to do I feel like she still circumvents that. And it’s very frustrating to me.”
	Removed child’s phone from their possession as punishment	7 (58)			
	Limited the time or times of the day their child can use the internet	8 (67)			
	Used parental controls to block certain content	7 (58)			
	Used parental controls to restrict phone use	4 (33)			
**Monitoring: covert or overt review of an adolescents’ DMU**			Open dialog about DMU builds or sustains trust in the parent-child relationship	Monitoring DMU was viewed as exhausting and only done when there was a reason for concern	“I have a program that when I asked them for their phones, I could plug it in to my computer, and I could get all their information on their phones. What I was really interested in was their text messages and their notes because in their notes is where they would write things that were revealing about their safety and also what websites they go to.”
	Checked which websites the child visited	9 (75)			
	Checked child’s profile on social media	10 (83)			
	Used monitoring tools to track location	9 (75)			
	Discussed appropriate web-based behavior	11 (92)			

^a^DMU: digital media use.

#### Clinician Group Discussion

A group discussion was conducted with 10 clinicians from different disciplines (6 mental health counselors, 2 clinical social workers, and 2 psychiatric nurses). Participants were predominantly female (7/10, 70%) and ranged in age from 27 to 63 years (mean 32.8, SD 11.8). During the group discussion, clinicians described having a role in discussing DMU with parents and adolescents. To reduce adolescents’ negative experiences with DMU, clinicians helped patients consider reducing access to upsetting, triggering, or bullying content, and omitting use during sleep times. Further, they promoted healthy digital expression by suggesting privately contacting a support person rather than publicly broadcasting suicidal thoughts. Clinicians who incorporated positive experiences with DMU as part of the treatment primarily discussed the use of DMU for distraction and social support. Clinicians felt that their role with parents involves education about DMU and its influence on youth mental health as well as helping both parents and adolescents navigate compromises with monitoring:

We often have to talk to families about finding that middle path, because they—parents want their children to go to them. But their kids aren’t gonna go to them if they know that their cell phone is gonna be taken away.

### Perceptions on Automated Digital Media Monitoring

#### Proposed Strategy for Automated Digital Media Monitoring

We described our idea for a 3-step process for the automated monitoring of adolescents’ DMU to participants and asked them to offer feedback. In step 1, adolescents and parents are securely connected to a website on which their permission would be requested to release the adolescents’ digital media content from several platforms. In step 2, the software automatically detects language indicative of suicidal risk from the adolescents’ digital media data. Finally, in step 3, when risk is detected, an alert is automatically sent to the adolescents’ clinician for response, as deemed necessary.

#### Parent and Adolescent Perspectives

Parents and adolescents identified facilitators and barriers associated with automated monitoring.

##### Protection From Harm

Parents and adolescents reflected that the chief facilitator is the potential for automated monitoring to protect digital media environments. Parents reflected on their capacity to identify suicidal risks. Despite their existing monitoring strategies, some parents felt that it was difficult to know when their child had suicidal thoughts. They believed that the automated detection of risk could aid their ability to maintain their child’s safety. Several adolescents felt that automated monitoring could protect them from harm, particularly on anonymous sites where they felt victimization is more frequent. Furthermore, some youth described that more adolescents are inclined to disclose suicidal thinking on digital media, who may not do so in person to a key support person who could act to prevent a crisis. They felt that automated monitoring has the potential to detect youth who reach out for help through digital media when their comments may otherwise go unnoticed:

I’m sure that a lot of kids turn to social media, because they don’t know how to turn to the people in real life. And sometimes it’s easier hiding behind a screen.

adolescent

##### Automated Risk Detection

Adolescents and parents were generally accepting of monitoring strategies that used software to detect suicidal risk. Adolescents consistently found the use of software acceptable because automation would reduce the private information received by clinicians to only content indicative of suicidal risk. Several parents appreciated that the automated detection of risk would decrease the burden associated with the manual review of their child’s digital media content. They also felt that the use of software would result in a greater reach than what they were capable of on their own:

That’s an awesome idea...if you had a way to monitor it sort of automatically then I feel like that would be more instrumental in finding out what’s really going on. You know? Like, I can only do what I can see, what she’ll let me see on her phone.

parent

##### Involvement of Clinicians

Parents and adolescents appreciated that automated monitoring would prompt conversations with clinicians about the risk of DMU. All parents saw the involvement of a trained mental health professional as a benefit. They felt that clinicians are likely to be effective because they regularly engage with their children and know their circumstances well. Some parents had experience working with clinicians who addressed DMU during treatment, which they felt helped their child gain insight. Although adolescents agreed that these conversations may be difficult, they acknowledged that being directly asked about risk language would help them engage with their clinician about their digital environments:

Yeah, especially for like the people that post on social media stuff like they post about their self-harm. I think that is really good to refer that to a therapist because not only is it somebody like looking this up out of curiosity or speculating that this person needs help, because it’s like proof that they do.

adolescent

##### Loss of Digital Privacy

Parents and adolescents agreed that their primary concern is the loss of privacy associated with releasing DMU for automated monitoring. Most adolescent and parent participants feared the release of personal communications on digital media, particularly sensitive text messages:

You know, I guess with social media I would be a little more comfortable just because it’s...already out there anyway. I think I [feel] more adversely at the text side.

parent

##### Potential for False Labeling

Due to the complicated ways in which adolescents communicate through DMU, many did not fully trust the ability of software to detect risk. Adolescents were concerned about whether a machine could effectively interpret sarcasm pertaining to suicidal communication:

Some people are serious, some people are just joking, some people are suicidal and joking. But there are so many jokes about wanting to kill yourself, that it would be too hard to actually pinpoint the actual people who are at risk.

adolescent

##### Tendency to Alter Behavior

When adolescents became aware that their DMU was being monitored, some adolescents suggested that they may alter their behavior. They suspected that some may change their behavior to negate the potential for risk alerts to be generated:

It would make people go off of it. They’d find their way around it. Or it’d be completely fake people trying to be happy so that they wouldn’t get monitored. But, at the same time, no monitoring is also kind of an issue.

adolescent

##### Communication With Parents About Risk

Most parents trusted their child’s therapist to gauge when they need to be made aware of risk alerts but acknowledged the need for a protocol to alert parents to safety risks:

I would hope that they would at least send a text message and let me know that there’s possibly a problem...I mean, if my child’s in danger, yeah...I want to know.

parent

#### Clinicians’ Perspectives

Through the group discussion, clinicians worked together to find a consensus on the risks and benefits of the proposed automated monitoring approach.

##### Potential Risks

Clinicians’ chief concern was their liability to respond when receiving risk alerts. Clinicians were concerned about not having the capacity to respond at all hours. They felt it was critical to have a feasible safety protocol in place and to assure patient and parent buy-in before proceeding with this monitoring approach. In addition, clinicians acknowledged the concerns that automated monitoring may not be compatible with all patients, specifically those who may have deficits in communication. One clinician considered potential challenges in identifying the risk in patients with autism:

I’ve had three different kids over the years who have been autistic. They would say something, and then they were done with it. But then all these fire alarms went off, metaphorically. [Mobile crisis support] was called...and the kid says “uh, I’m fine.”

##### Accommodations

Clinicians suggested accommodations to the automated monitoring strategy to reduce liability. They considered the potential for parents to be alerted to risk language instead of clinicians. However, some clinicians felt that this could result in less buy-in from adolescents who may not want their parents to have that level of information and access to their DMU. Consensus was reached upon an alternative in which adolescents themselves receive alerts when risk is detected and are automatically provided with feedback on how to respond based on their safety plan:

I think that would almost be the best way to do this because then you’re creating the awareness for them in the moment, that, “oh wait, hey, I’m at higher risk right now,” or “I’m feeling worse, and I’m saying these things, this might be a great time for me to utilize coping skills or reach out for support.”

In addition to youth receiving in-the-moment alerts, clinicians wanted to be informed of trends regarding risk detected through adolescents’ DMU. They suggested one option would be to provide an index of suicide risk severity over time, that they could review with youth during therapy sessions.

##### Potential Benefits

If the automated monitoring strategy was revised to meet their needs, clinicians felt it could have advantages both for them and their patients. Clinicians felt that this would aid their ability to monitor patients’ symptoms over time. Furthermore, clinicians felt that an approach alerting adolescents to the potential for risk would allow autonomy in managing their mental health, similar to how patients manage their physical health:

I think it kinda reminds me...of someone who has diabetes and is like checking your blood sugar to say like, “Oh, I need to take my insulin.” It lets them have control.

## Discussion

### Principal Findings

This study offers insights from parents, adolescents, and mental health clinicians to inform the development of an automated method for digital media monitoring aimed at detecting and responding to adolescent suicidal risk. All participants reported challenges to the current monitoring approaches. Although adolescents perceived the negative consequences of DMU on their mental health, they were displeased by parental monitoring and experienced discomfort in starting a conversation with their therapists about DMU. Parents expressed challenges in monitoring their children’s DMU, which left them feeling powerless. Clinicians felt that they had a role in managing parent-child relationship dynamics pertaining to digital media monitoring. Participants perceived that automated monitoring has a potential for advantage beyond the current monitoring approaches but had concerns that could act as barriers to implementation.

The results of this study suggest a need to honor adolescents’ desires for free and private expression that could aid engagement with supportive peers, while also honoring parents’ need to protect adolescents’ safety. Clinicians and adolescents reported that younger children, in particular, require protection. Their assertions are consistent with recommendations from UNICEF (United Nations International Children’s Emergency Fund), which suggests the need to temper approaches to monitoring based on a child’s developmental capacity [24], and the American Association of Suicidology [25], which suggests determining a monitoring approach based on adolescents’ individual vulnerability to the harmful aspects of digital media as well as its benefits. When adolescents are suicidal, monitoring intensity should be consistent with their level of maturity, acuity, and risk of self-harm.

The results from this study also suggest the need to screen for DMU as part of clinical care. Exemplifying this point, clinicians in this study felt that they openly discussed DMU with their adolescent patients, whereas adolescents rarely reported talking about DMU with their clinicians. Although it is possible that these differences in report may have been influenced by the 1.5-year gap in data collection between adolescents or parents and clinicians, the clinical care and management of concerns related to DMU in this setting remained stable within this period. Furthermore, other data have shown adolescents’ hesitance in discussing DMU with trusted adults. For example, only 11% of adolescents reported disclosing incidents of cyberbullying [26]. Using a validated questionnaire, such as the Problematic and Risky Internet Use Screening Scale [27,28], as part of the clinical assessment could aid adolescents’ discomfort in initiating a discussion on DMU and support an open line of communication that will aid informed suicide risk management.

**Figure 1:**
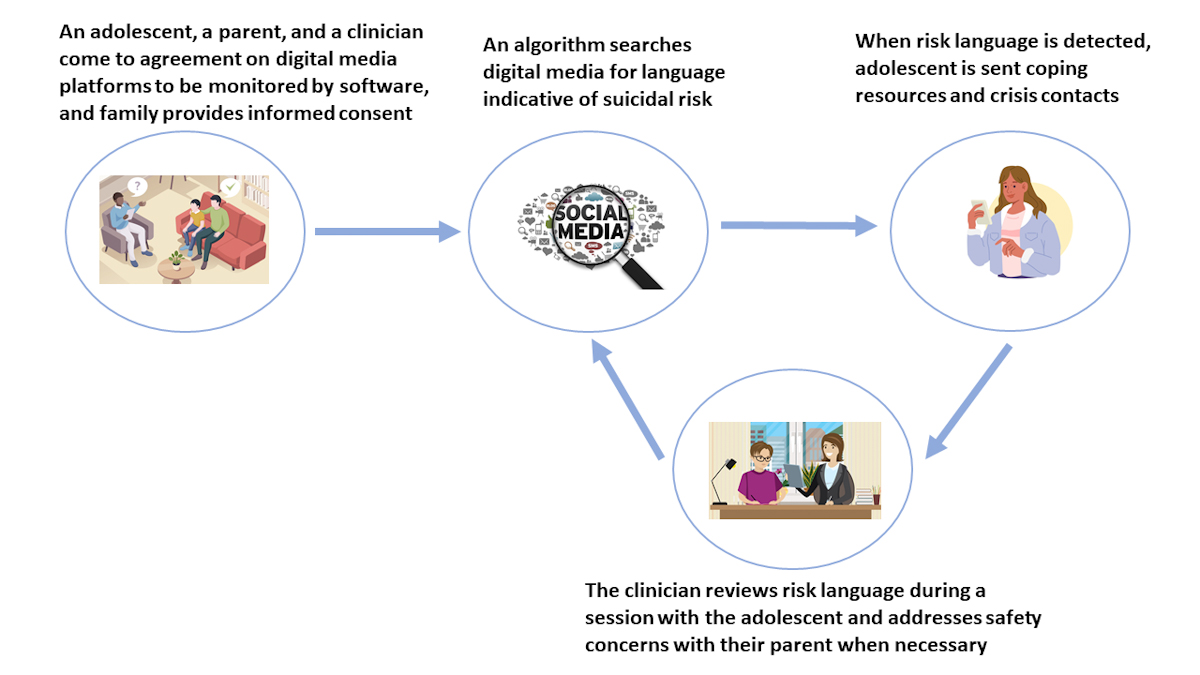
Final strategy for automated digital media monitoring based on participant feedback.

Participants’ perceptions of the facilitators and barriers of automated monitoring suggest the need for an approach that is responsive to their preferences. This approach could leverage adolescents’ and parents’ desires for a protected digital environment to garner interest. To mitigate hesitance toward the release of private information, flexibility is recommended whereby adolescents and parents can decide what information they are willing to share. The role of clinicians is critical in explaining the potential risks before proceeding. Discussion regarding risks should recognize the potential for false alerts, that is, the possibility that automated analysis could misinterpret ambiguous language as risky or that indications of risk could be missed, while also recognizing NLP’s potential to detect subtle communications of suicidal risk [14,15]. Furthermore, clinicians requested accommodations to the proposed method of automated monitoring. They desired alerts of risk language to be delivered to adolescents, alongside coping resources and crisis contacts from adolescents’ safety plans. This approach could innovatively address the guidance by the American Association of Suicidology to incorporate the positive and negative aspects of DMU into safety planning for youth at risk for suicide [25] through novel automated methods. A revised strategy that consolidates the participants’ recommendations is shown in Figure 1. Although this revised approach should be further reviewed with adolescents and parents, it is likely to offer adolescents autonomy in their communication through DMU, a key need that they identified for digital media monitoring. Furthermore, this revised approach would retain clinician involvement in monitoring through the adolescents’ clinical care, which was desirable to parents.

Next steps will include the use of human-centered design, a methodology known to enhance the implementation of digital mental health interventions [29], to develop prototypes of a clinician dashboard and interface to provide youth with coping and crisis resources when risk is indicated. Subsequently, we will test this approach using an evidence-based NLP algorithm. This design and testing process will also include additional iterative steps with adolescents and parents to optimally address the barriers they have identified, namely, those related to privacy and the risk of false positives, while harnessing facilitators toward engagement in automated monitoring.

### Strengths and Limitations

This study had several limitations. First, the use of a convenience sample, the small sample size, and the exploratory nature of this study limit our ability to generalize our results to a larger population. Participants were part of an IOP at an academic medical center; therefore, our results may differ from those obtained from other settings and may not be representative of adolescents who do not seek mental health care. Additional perspectives are necessary to generalize to settings with differing levels of resources or for adolescents and families who do not seek clinical care. Furthermore, there was a lag in data collection of adolescents and parents in 2018 and clinicians in 2020. When interpreting clinicians’ perspectives in the context of parents’ and adolescents’ reports, readers should acknowledge the potential that clinicians’ awareness of digital media’s influence on adolescents may have changed within this period. Despite these limitations, this study offers insights into the current context of monitoring of suicidal adolescents’ DMU and provides a breadth of information that will fuel an acceptable approach to the automated monitoring of high-risk youth, thereby strengthening its feasibility for implementation in clinical practice.

### Conclusions

Our findings provide valuable insights into the development of a feasible automated monitoring intervention that can be implemented in the clinical care of suicidal youth. Involving adolescents, parents, and clinicians in the development of approaches for automated monitoring is likely to result in a more widely accepted, understood, and effective monitoring strategy and a greater capacity to protect adolescents from harmful DMU experiences.

## Abbreviations

DMU: digital media use
IOP: intensive outpatient program
NLP: natural language processing
UNICEF: United Nations International Children’s Emergency Fund

## References

[1] Hedegaard H, Curtin SC, Warner M (2018). Suicide mortality in the United States, 1999-2017. NCHS Data Brief.

[2] Marchant A, Hawton K, Stewart A, Montgomery P, Singaravelu V, Lloyd K, Purdy N, Daine K, John A (2017). A systematic review of the relationship between internet use, self-harm and suicidal behaviour in young people: the good, the bad and the unknown. PLoS One.

[3] Dyson MP, Hartling L, Shulhan J, Chisholm A, Milne A, Sundar P, Scott SD, Newton AS (2016). A systematic review of social media use to discuss and view deliberate self-harm acts. PLoS One.

[4] Viner RM, Gireesh A, Stiglic N, Hudson LD, Goddings A, Ward JL, Nicholls DE (2019). Roles of cyberbullying, sleep, and physical activity in mediating the effects of social media use on mental health and wellbeing among young people in England: a secondary analysis of longitudinal data. Lancet Child Adolesc Health.

[5] Biernesser C, Bear T, Brent D, Mair C, Trauth J (2019). Social media and adolescent suicide: exploring risks, benefits, and opportunities for prevention. University of Pittsburgh, PhD Dissertation.

[6] Hamm MP, Newton AS, Chisholm A, Shulhan J, Milne A, Sundar P, Ennis H, Scott SD, Hartling L (2015). Prevalence and effect of cyberbullying on children and young people: a scoping review of social media studies. JAMA Pediatr.

[7] Spada MM (2014). An overview of problematic internet use. Addict Behav.

[8] Liu H, Liu S, Tjung J, Sun F, Huang H, Fang C (2017). Self-harm and its association with internet addiction and internet exposure to suicidal thought in adolescents. J Formos Med Assoc.

[9] Belfort EL, Miller L (2018). Relationship between adolescent suicidality, self-injury, and media habits. Child Adolesc Psychiatr Clin N Am.

[10] Jacobs D, Baldessarini R, Conwell Y, Fawcett J, Horton L, Meltzer H, Pfeffer CR, Simon RI (2010). Practice guidelines for the assessment and treatment of patients with suicidal behaviors. American Psychiatric Association.

[11] Franklin JC, Ribeiro JD, Fox KR, Bentley KH, Kleiman EM, Huang X, Musacchio KM, Jaroszewski AC, Chang BP, Nock MK (2017). Risk factors for suicidal thoughts and behaviors: a meta-analysis of 50 years of research. Psychol Bull.

[12] Rideout VR, Robb MB (2018). Social media, social life: teens reveal their experiences. Common Sense Media.

[13] Nadkarni PM, Ohno-Machado L, Chapman WW (2011). Natural language processing: an introduction. J Am Med Inform Assoc.

[14] Coppersmith G, Leary R, Crutchley P, Fine A (2018). Natural Language Processing of Social Media as Screening for Suicide Risk. Biomed Inform Insights.

[15] De Choudhury M, Kiciman E, Dredze M, Coppersmith G, Kumar M (2016). Discovering shifts to suicidal ideation from mental health content in social media. Proc SIGCHI Conf Hum Factor Comput Syst.

[16] Patton MQ (2001). Qualitative Research & Evaluation Methods. 3rd Ed.

[17] Payne G, Payne J (2004). Group Discussions/Focus Groups.

[18] Anderson M (2016). Parents, teens and digital monitoring. Pew Research Center.

[19] Clandinin D, Connelly F, Denzin N, Lincoln Y (1994). Personal experience methods. Handbook of Qualitative Research.

[20] Morse JM (2016). The significance of saturation. Qual Health Res.

[21] (2018). NVivo qualitative data analysis software, version 12. QSR International Pty Ltd.

[22] Braun V, Clarke V (2006). Using thematic analysis in psychology. Qual Res Psychol.

[23] Braun V, Clarke V (2014). What can "thematic analysis" offer health and wellbeing researchers?. Int J Qual Stud Health Well-being.

[24] (2018). Children's online privacy and freedom of expression. UNICEF.

[25] (2019). Suicide and social media. American Association of Suicidology.

[26] (2010). Stop cyberbullying before it starts. National Crime Prevention Council.

[27] Moreno MA, Arseniev-Koehler A, Selkie E (2016). Development and testing of a 3-item screening tool for problematic internet use. J Pediatr.

[28] Jelenchick LA, Eickhoff J, Christakis DA, Brown RL, Zhang C, Benson M, Moreno MA (2014). The Problematic and Risky Internet Use Screening Scale (PRIUSS) for adolescents and young adults: scale development and refinement. Comput Human Behav.

[29] Mohr DC, Lyon AR, Lattie EG, Reddy M, Schueller SM (2017). Accelerating digital mental health research from early design and creation to successful implementation and sustainment. J Med Internet Res.

